# Reply to Hernández—Glycolysis and gluconeogenesis: A teaching view

**DOI:** 10.1016/j.jbc.2020.100021

**Published:** 2020-12-08

**Authors:** Ankit M. Shah, Fredric E. Wondisford

**Affiliations:** Department of Medicine, Robert Wood Johnson Medical School, Rutgers University, New Brunswick, New Jersey, USA

We thank Félix Hernández for his insightful and thoughtful comments as well as interest in our manuscript (1). Regarding suggestion 1, we omitted several glycolytic and gluconeogenic enzymes in the figure for simplicity. We specifically mentioned G6Pase and PEPCK as many investigators have studied these enzymes in particular when studying gluconeogenesis. Articles specifically referenced in our review article studied these two enzymes, and we wished to pictorially represent these enzymes. We further described each of these two enzymes’ specific functions in the text. Our manuscript does not go into the regulation of glycolysis or gluconeogenesis as our main intention was to show carbon flux. So we do not feel compelled to label the enzymes as suggested as that is not the intent of the picture.

Regarding suggestions 2 and 3, we agree that there should be a single arrow between phosphoenolpyruvate to pyruvate and there should be a single arrow between pyruvate to oxaloacetate to best show carbon flux (Fig. 1).

**Figure 1:**
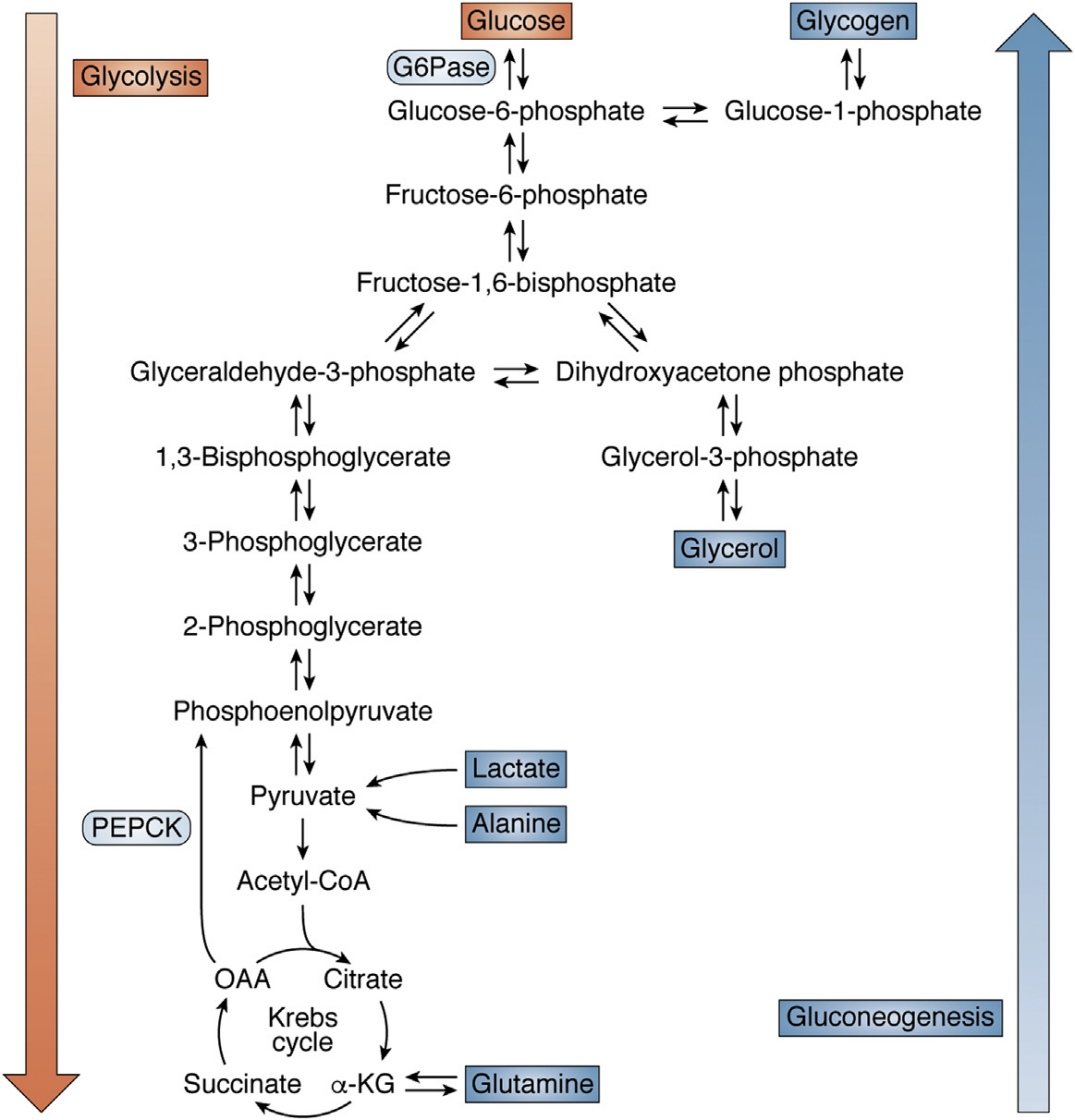
**Glucose metabolism in the context of glycolysis and gluconeogenesis.** α-KG, alpha-ketoglutarate; G6Pase, glucose-6-phosphatase; OAA, oxaloacetate; PEPCK, phosphoenolpyruvate carboxykinase.

## Conflict of interest

The authors declare that they have no conflicts of interests with the contents of this article.
